# Ischaemic stroke-induced distal organ damage: pathophysiology and new therapeutic strategies

**DOI:** 10.1186/s40635-020-00305-3

**Published:** 2020-12-18

**Authors:** Chiara Robba, Denise Battaglini, Cynthia S. Samary, Pedro L. Silva, Lorenzo Ball, Patricia R. M. Rocco, Paolo Pelosi

**Affiliations:** 1 Anesthesia and Intensive Care, San Martino Policlinico Hospital, IRCCS for Oncology and Neuroscience, Largo Rosanna Benzi 10, 16100 Genoa, Italy; 2 Department of Surgical Sciences and Integrated Diagnostics, University of Genoa, Genoa, Italy; 3 Laboratory of Pulmonary Investigation, Carlos Chagas Filho Institute of Biophysics, Federal University of Rio de Janeiro, Rio de Janeiro, Brazil

**Keywords:** Stroke, Extracranial complications, Cardiac dysfunction, Organs cross talk, Stroke-associated pneumonia

## Abstract

Acute ischaemic stroke is associated with a high risk of non-neurological complications, which include respiratory failure, cardiovascular dysfunction, kidney and liver injury, and altered immune and endocrine function. The aim of this manuscript is to provide an overview of the main forms of stroke-induced distal organ damage, providing new pathophysiological insights and recommendations for clinical management.

Non-neurological complications of stroke can affect outcomes, with potential for serious short-term and long-term consequences. Many of these complications can be prevented; when prevention is not feasible, early detection and proper management can still be effective in mitigating their adverse impact. The general care of stroke survivors entails not only treatment in the acute setting but also prevention of secondary complications that might hinder functional recovery. Acute ischaemic stroke triggers a cascade of events—including local and systemic activation of the immune system—which results in a number of systemic consequences and, ultimately, may cause organ failure. Understanding the pathophysiology and clinical relevance of non-neurological complications is a crucial component in the proper treatment of patients with acute stroke.

Little evidence-based data is available to guide management of these complications. There is a clear need for improved surveillance and specific interventions for the prevention, early diagnosis, and proper management of non-neurological complications during the acute phase of ischaemic stroke, which should reduce morbidity and mortality.

## Background

**Fig. 1:**
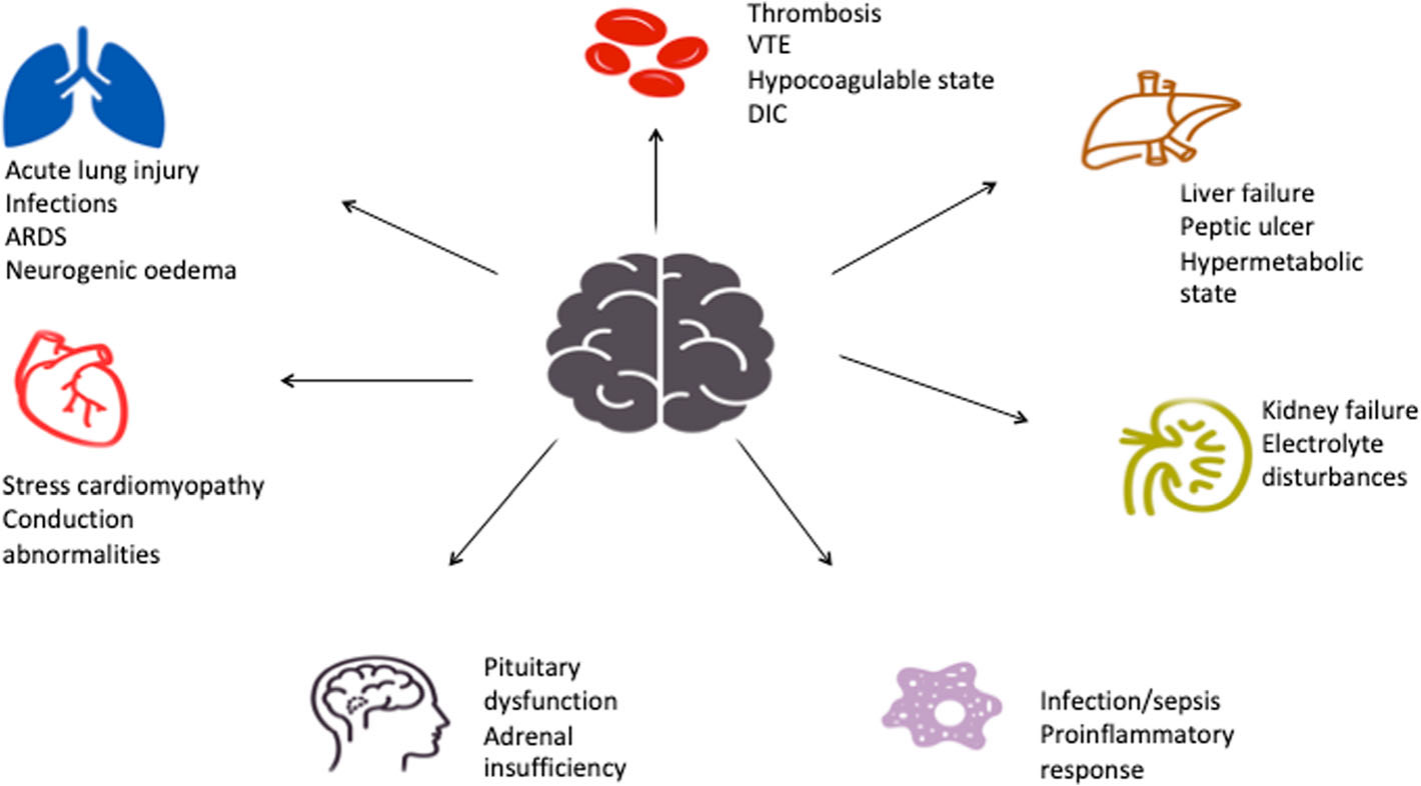
Main types of peripheral organ dysfunction after stroke

The brain controls various body functions through complex neurohumoral mechanisms. Therefore, any severe cerebral insult, such as that which follows an acute ischaemic stroke, can induce several changes in specific neurosensory or neuromotor pathways, enhance the systemic response to local injury, and cause secondary peripheral organ damage [1]. Although the incidence of ischaemic stroke is declining, it remains the second leading cause of death worldwide [2]. Amongst stroke subtypes, ischaemic stroke is the most frequent, though incidence significantly varies across countries [2]. Current research suggests that only 28% of stroke patients have a favourable outcome (assessed through the modified Rankin scale) at 3 months [3]. The high risk of neurological deterioration and systemic organ dysfunction after stroke often requires intensive care unit admission, consequently increasing hospitalisation costs [4]. Overall, critically ill patients are particularly prone to developing organ dysfunction. The multiple organ dysfunction syndrome (MODS) occurs in up to 14% of these patients, and accounts for 80% of intensive care unit deaths [5]. Few studies have reported data on systemic complications after stroke, but the incidence of MODS is estimated at up to 12% of cases [6] (Figure 1). Low Glasgow Coma Score, advanced age, hypo- and hyperglycaemia, leucocytosis, and a history of chronic disease have been identified as risk factors for MODS after stroke [6]. Although some experimental data on stroke-induced peripheral organ damage and MODS is available, the literature on clinical features of multiorgan failure and its management after stroke is scarce. Therefore, the aim of our manuscript is to describe the prevalence, pathophysiology, features, and treatment of the main types of distal organ damage occurring after acute ischaemic stroke. For this purpose, we searched four electronic databases (PubMED, Scopus, ScienceDirect, Web of Science) for manuscripts describing extracranial complications after acute ischaemic stroke, with no time restriction. Only manuscripts in English were considered; titles and abstracts were retrieved and independently assessed for eligibility by two authors (CR, DB). Disagreements were resolved by discussion and consensus agreement, and, if required, input from a third author (CSS).

## Respiratory complications

### Brain-lung crosstalk in stroke

**Fig. 2:**
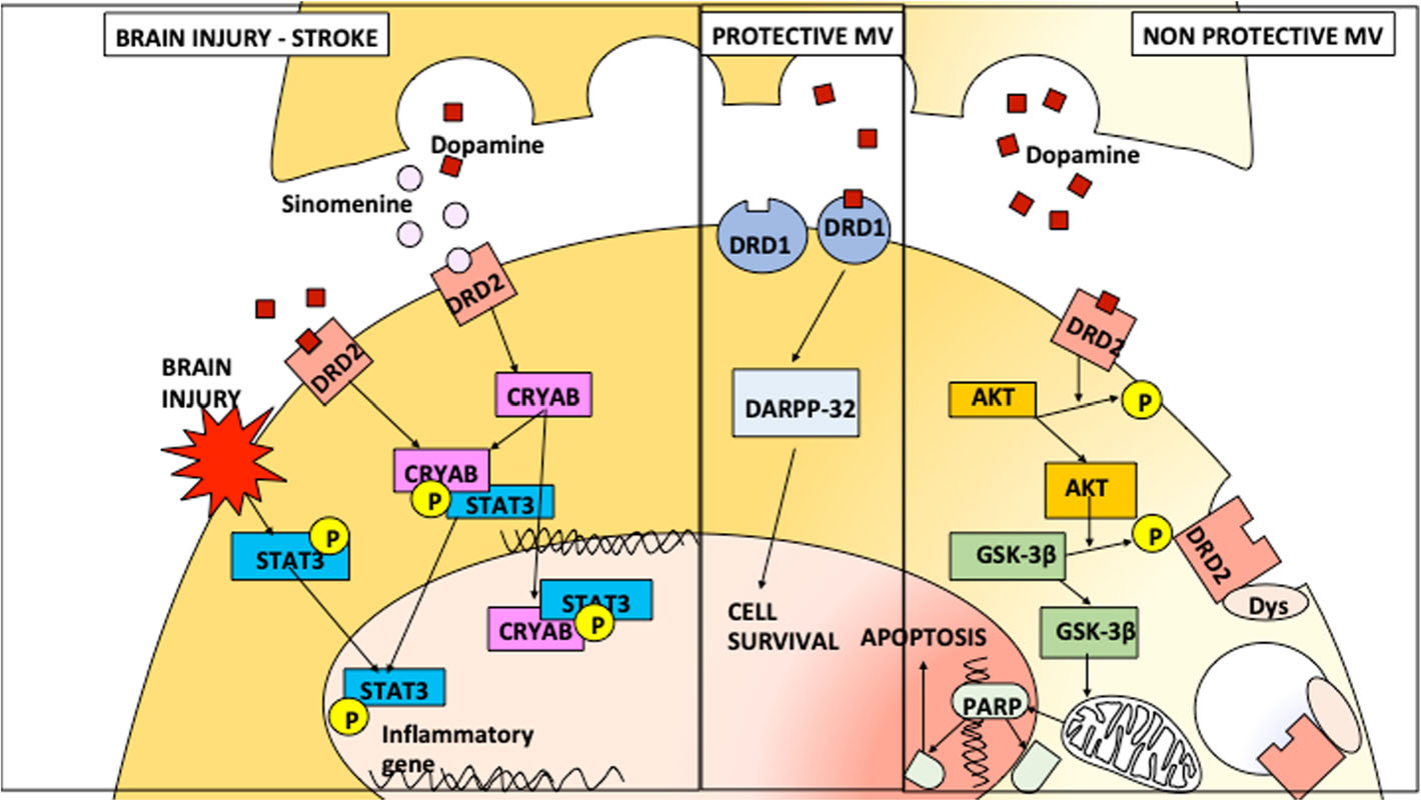
Mechanisms of immunomodulation and cell apoptosis induced by the dopamine pathway. Under normal conditions, dopamine activates type 1 receptors (DRD1), promoting cell survival. The increased release of dopamine triggered by non-protective mechanical ventilation activates type 2 receptors (DRD2), triggering a cascade of events resulting in apoptosis. Activation of astrocytic DRD2 induces upregulation and nuclear translocation of the αB-crystallin CRYAB/STAT3 pathway, which alleviates neuroinflammatory injury by reducing the generation of pro-inflammatory cytokines. In this context, dopamine agonists and antagonists can play a role in regulating the neuroinflammatory response; in particular, haloperidol—which competitively blocks postsynaptic dopamine receptors in the mesolimbic dopaminergic system—has different effects on cell survival depending on the use of protective and non-protective ventilation. Sinomenine can blunt the CRYAB/STAT3 pathway by acting on DRD2 receptors, thus suppressing neuroinflammation

Data suggest that pulmonary complications are common and occur early after stroke, are common causes of intensive care unit admission, and are associated with high mortality and morbidity rates [7]. The lung is particularly susceptible to severe brain damage (such as that which follows ischaemic and haemorrhagic stroke). A recent clinical study found that, 36 h after hospital admission, 15.6% of stroke patients had acute lung injury, and 7.8% developed pneumonia or bronchitis during their hospital stay [8]. Different pathways have been implicated in *brain-lung crosstalk* after stroke [9]. Experimental stroke models have focused mostly on the role of immune modulation, as local and systemic immune activation is known to predispose to lung injury [10]. Although clinical trials in this particular subpopulation of intensive care unit patients are lacking, the use of protective ventilation strategies and administration of dopamine agonists and antagonists to minimise brain and lung damage has been suggested in experimental settings [10, 11] (Fig. 2).

### Acute respiratory distress syndrome and hypoxia

Among respiratory complications [12, 13], the acute respiratory distress syndrome (ARDS) is a severe pulmonary condition characterised by severe hypoxia and bilateral lung infiltrates [14]. In a large retrospective cohort study of patients with acute ischaemic stroke, ARDS prevalence was found to be low (about 4%), but when it did occur, it had a significant impact on in-hospital mortality [14]. This incidence seems to be lower than in other brain-injured groups; e.g., ARDS occurs in up to 30% of patients with subarachnoid haemorrhage [15]. A recent experimental study investigated ARDS incidence in stroke and found that focal ischaemic stroke was often followed by acute lung injury, but not by full-blown ARDS [16]. Aspiration of oropharyngeal and gastric content is one of the main causes of ARDS, occurring in up to 3.6% of stroke patients, and it is associated with swallowing dysfunction, neurologic dysphagia, and gastrointestinal issues [17].

The incidence of ARDS in brain-injured patients is decreasing [18]. Overall, the neurologic population is shifting from one of predominantly traumatic brain injury to one of non-traumatic cerebral haemorrhage and ischaemic stroke, which is likely to influence future clinical, diagnostic, and research practise in health care systems. The main issue in the management of ARDS patients affected by stroke is whether one should apply a protective ventilator strategy but maintain normocapnia (with an arterial partial pressure of carbon dioxide between 35–45 mmHg) [19]. Indeed, although permissive hypercapnia is commonly used in patients with ARDS, it can have a detrimental effect on cerebral blood flow and intracranial pressure [19]. Current ventilatory strategies to manage ARDS have been investigated in multicentre studies conducted in non-brain-injured patients; none has focused on stroke patients as a subpopulation. In the general population, evidence suggests the use of low tidal volumes (6 mL/kg predicted body weight [PBW]) to reduce mortality and increase ventilator-free days [20]. Likewise, recruitment manoeuvres (RM) and positive end-expiratory pressure (PEEP) are considered part of any protective ventilation strategy [20], but may increase intrathoracic pressure and impair cerebral perfusion pressure [21].

Hypoxia with arterial oxygen saturation < 90% in the first hours after hospital admission is associated with a twofold risk of death [22]. However, recent evidence suggests that hyperoxia must be avoided as well, because it is associated with high mortality, delayed cerebral ischaemia, respiratory tract infections, and poor outcomes in brain-injured patients [23–25]. A recent meta-analysis showed that hyperoxia may be associated with increased mortality in different groups of patients, including after stroke [24].

No specific recommendations are available for stroke patients with ARDS; maintaining an arterial pressure of oxygen between 60–80 mmHg in both ARDS and brain-injured patients is widely accepted [19]. The fraction of inspired oxygen (FiO_2_) should be titrated to achieve the minimal acceptable O_2_ saturation (SpO_2_) (88–92%). Driving pressure should be kept < 13 cmH_2_O, and respiratory rate as low as possible for pH > 7.25 [26].

### Stroke-associated pneumonia and lower respiratory tract infection

Pneumonia is one of the most common complications after stroke, largely due to aspiration consequent to the inability of stroke patients to maintain airway patency [27]. However, the incidence of pneumonia after stroke is higher with respect to general (non-stroke) intensive care unit patients who suffer from dysphagia and altered level of consciousness, thus suggesting a role of causative mechanisms other than aspiration, such as abnormal immune patterns and lung dysfunction [28]. Additionally, the incidence of stroke-associated pneumonia is higher in intensive care units than in non-intensive stroke wards, ranging from 3.9–56.6% [29]. Common independent risk factors for aspiration pneumonia in stroke patients were recently investigated in the PREDICT multicentre observational study, which confirmed that both stroke-associated immunodepression and dysphagia can contribute to stroke-associated pneumonia [28]. Although several scores have been proposed for the prediction of stroke-associated pneumonia, their use in clinical practise is uncommon [30]. Biomarkers (such as C-reactive protein), stroke severity scores, and dysphagia scores remain most commonly used at bedside [31]. Early mobilisation is strongly recommended, while early antibiotic therapy seems not to improve functional outcome at 3 months in adults with acute ischaemic stroke [32].

### Ventilator management in non-ARDS stroke patients

Both mechanical and neurohumoral inflammatory mechanisms are involved in brain-lung crosstalk, enhancing the possible negative effects of mechanical ventilation on specific areas of the brain, even in patients without pre-existing respiratory disease [7]. Although large, multicentre clinical trials on mechanical ventilation settings have often excluded neurologic patients, several experimental studies have investigated this issue. Quilez et al. investigated three different respiratory strategies (mechanical ventilation with different tidal volumes versus spontaneous breathing) in healthy rats, demonstrating the development of significant morphofunctional and biochemical alterations in mechanically ventilated as compared to non-ventilated rats [33]. Additionally, in ventilated rats, the authors observed increased release of inflammatory markers in the lung and plasma, as well as *c-fos* gene activation in the central amygdala, hippocampus, paraventricular hypothalamic nuclei, and supraoptic nucleus. Rats ventilated with higher tidal volumes also exhibited a heightened inflammatory response, thus suggesting an important role of ventilator-induced lung injury on peripheral organ damage, including in the brain [33]. Furthermore, specific mechanisms have been implicated in lung injury after stroke. Samary et al. conducted an experimental study on rats which demonstrated increased diffuse alveolar damage (especially in endothelial cells and type II pneumocytes), pulmonary oedema, and inflammatory markers in the lungs of stroked rats. A heightened pro-inflammatory response was demonstrated in the brain, lung, plasma (tumour necrosis factor-α and interleukin-6), and bronchoalveolar lavage fluid (tumour necrosis factor-α) [34]. Moreover, in an experimental study on pigs with haemorrhagic stroke, high PEEP levels (> 20 cmH_2_O) caused impairment of autoregulation with decreased mean arterial pressure, but without impairing intracranial pressure, cerebral oxygenation, or regional cerebral blood flow [35]. Specific pathophysiological mechanisms involved in stroke and the effects of protective versus non-protective mechanical ventilation strategies are summarised in Fig. 2.

Optimal settings for mechanical ventilation in stroke patients without ARDS remain controversial [36]. Protective mechanical ventilation using a tidal volume of 6 mL/kg PBW, adequate PEEP levels, plateau pressure < 30 cmH_2_O, and recruitment manoeuvres (RMs) as needed has been associated with reduced mortality (up to 10%) in general (non-ARDS) critically ill patients [20]. However, a recent trial of non-ARDS patients (8% affected by brain injury) suggested that low tidal volume strategies (4–6 mL/kg) do not reduce ventilation-free days when compared to intermediate tidal volume strategies (8 mL/kg) [37]. Otherwise, a clinical trial of general brain-injured patients showed an improvement in ventilator-free days in those treated with a protective ventilator strategy (tidal volume of 6–8 ml/kg of PBW and PEEP > 3 cmH_2_O) [38]. Another multicentre trial which included more than 700 brain-injured patients demonstrated that protective mechanical ventilation (tidal volume < 7 ml/kg PBW, PEEP 6–8 cmH_2_O) can improve ventilator-free days at day 90 and reduce mortality rate [39]. A recent trial by Mascia et al. demonstrated that the application of PEEP increases intracranial pressure only in patients in whom PEEP causes alveolar hyperinflation and hypercapnia [40], whereas other authors have suggested that PEEP has a detrimental effect on cerebral haemodynamics only if it affects mean arterial pressure and cerebral perfusion pressure [41]. With regard to RMs, no conclusive evidence is available for non-ARDS patients, although these should not be used routinely; no evidence whatsoever is available for brain-injured patients in particular [21]. Taken together, these findings suggest that PEEP and RMs manoeuvres may have a role to play in mechanically ventilated stroke patients, but should only be performed under strict hemodynamic and neurologic monitoring [42]. In the absence of ARDS, we recommend using a protective tidal volume (6–8 mL/kg PBW), which can be increased up to 10 mL/kg PBW if needed; Pplat should be kept below 25 cmH_2_O, and PEEP 5 cmH_2_O in patients without ARDS (increased up to 15 cmH_2_O if necessary in ARDS).

## Cardiovascular complications

Common knowledge on cerebrovascular and cardiovascular diseases has been changing in recent last decades. Early detection of cardiovascular dysfunction directly caused by stroke, instead of independent cardiovascular illness, has become paramount. Researchers now generally agree on the existence of a bidirectional interaction between the brain and the heart [43].

Corroborating this so-called *brain-heart crosstalk* theory, stroke patients are extremely vulnerable to the development of severe cardiac complications, possibly due to changes in autonomic and neurohormonal pathways involved in the control of heart function [44]. Our understanding of the pathophysiology of stroke-heart interactions has improved greatly over the past few decades: inflammation and immunity [45], sympathetic hyperactivity [46], the hypothalamic-pituitary-adrenal axis [47], and gut dysbiosis [48] have been identified as the main pathologic factors involved in brain-heart axis dysregulation after both ischaemic and haemorrhagic stroke [46].

### Clinical implications

Data suggest that patients affected by stroke share the same cardiovascular risk factors of those affected by cardiovascular dysfunction [49, 50]. Most critical cardiovascular complications are identified within a few hours of stroke [51, 52]. The leading causes of mortality following acute stroke include heart attack [44], congestive heart failure, haemodynamic instability, left ventricular systolic dysfunction [53], diastolic dysfunction, arrhythmias [54], and cardiac arrest [44], which are all associated with delayed cerebral ischaemia, poor outcome, and death.

### Acute cardiac dysfunction

Acute cardiac dysfunction occurs in up to 67% of patients with ischaemic stroke [55]. Common manifestations include arrhythmias, electrocardiographic (ECG) changes [49], and acute myocardial infarction-like complications [56].

ECG changes occur in 15 to 30% of patients with stroke [57–59]. Frequent abnormalities include T wave inversion, ST elevation, ST depression, upright T waves, and pathological Q waves [55, 60].

Taking into account ischaemic myocardial damage, 50% of stroke patients without previous cardiac history suffers from coronary stenosis, with a high risk of myocardial infarction within the following 3 years [61]. A meta-analysis including 67,000 patients demonstrated that the annual risk for developing myocardial infarction after stroke is about 2%, followed by a 20% 10-year risk of myocardial ischaemia [60, 62]. A common myocardial infarction-like manifestation is neurogenic stunned myocardium, which differs from traditional myocardial damage because of the absence of coronary artery occlusion [63].

### Chronic cardiac dysfunction

A common consequence of arrhythmic disease and myocardial dysfunction is chronic myocardial remodelling [64, 65]. An experimental study in stroke mice confirmed the utility of beta blockers to decelerate extracellular cardiac remodelling by modulating sympathetic activity [66].

Diastolic and systolic ventricular dysfunctions are significant post-stroke complications, which affect 15–25% of patients [67]. Amongst these, in a retrospective cohort, 46% became functionally dependent or died [68].

### Treatment

Intensive care unit admission with continuous haemodynamic monitoring has been proposed as mandatory for patients with stroke at high risk of developing cardiovascular complications [69], based on the National Institute of Health Stroke Scale (NIHSS) [69]. Suggested haemodynamic monitoring for these patients includes ECG, continuous recording of vital signs, and echocardiography [68, 70, 71]. Some recent trials investigated the potential effect of specific therapies to prevent secondary cerebrovascular and cardiovascular accidents after stroke [72, 73]. Amongst these, drugs such as labetalol, nicardipine, and nitroprusside have been recommended to control hypertension during the acute phase of stroke [72], when systolic blood pressure exceeds 180–200 mmHg, while angiotensin-converting-enzyme inhibitors and angiotensin II receptor blockers are advised for the management of chronic hypertension [73]. A neuroprotective effect of propranolol has been recently demonstrated in experimental settings [11], and beta blockers have been suggested both for preventing chronic remodelling and for treatment of arrhythmias [73]. In addition, any electrolyte imbalances should be taken into account when rhythm abnormalities are treated [72].

In conclusion, therapeutic targets in stroke-related cardiovascular complications remain challenging. Further experimental and clinical investigations are essential to complete this complex multi-disciplinary puzzle.

## Other complications

### Renal complications

Acute kidney injury (AKI) affects approximately 35% of patients admitted to intensive care units [74–78]. In a recent meta-analysis, the pooled incidence of AKI was 9.6% in ischaemic stroke and 19.2% in haemorrhagic stroke [74]. Risk factors for AKI amongst studies varied significantly, and all authors concluded that AKI after stroke significantly increases mortality rate (OR 2.45) [74].

A large, multicentre study of a cohort of ischaemic stroke patients, 35% affected by AKI, found that AKI occurrence was associated with a higher risk of neurological deterioration and in-hospital mortality (138% greater) [79]. In addition, the risk of cardioembolic stroke was greatly increased in patients with AKI, as was the risk for recurrence, of which albuminuria was an independent predictor [80]. Albuminuria was also found to be a predictor of haemorrhagic transformation of ischaemic stroke [81]. Creatinine levels, in turn, were associated with large baseline hematoma volume [82], death, and severe disability [83] at 1 year.

### Hepatic complications

Following acute ischaemic stroke, glucose metabolism, and proteins implicated in insulin and growth hormone signalling in the liver are altered. Stroke-induced liver injury and hepatocyte dysfunction are associated with metabolic derangements, a decrease in hepatic transcription factors, and impaired host immune function, protein synthesis, and clearance of activated clotting factors [84]. Liver failure is an important risk factor for cardiovascular disease, but its association with cerebrovascular diseases such as ischaemic and haemorrhagic stroke is still poorly investigated [84]. In a recent study, approximately 61% of patients with no history of previous hepatobiliary dysfunction had abnormal hepatic enzyme levels at general intensive-care unit admission, and this finding was associated with increased short-term mortality.

The prevalence of hypoxic hepatitis is estimated at 1-12% in general critically ill patients [85]. Hypoxic hepatitis is a common consequence of cardiovascular insults due to inadequate oxygen delivery to distal organs, causing a tenfold elevation of aspartate aminotransferase, rise in international normalised ratio, and impaired renal function [85]. However, its association with stroke has not been studied to date [85].

### Endocrine complications

Current evidence suggests that anterior pituitary deficiency occurs in 19% of patients after ischaemic stroke [86]. A prospective, single-centre trial investigating hypopituitarism after stroke suggested that its incidence increases over time, and is associated with impaired functional outcome [86]. The same authors conducted another study to assess the risk of outcome severity in stroke patients with growth hormone and insulin-like growth factor-1 dysfunction [87]. Approximately 12% of patients exhibited growth hormone deficiency, while comparatively few developed hypogonadisms. Insulin-like growth factor and growth hormone levels showed a strong association with functional and neurocognitive outcomes [87].

Amongst anterior pituitary dysfunctions, adrenal insufficiency seems to be the most common, and has been identified in 31% of stroke patients [88]. However, the effect of adrenal insufficiency on outcome is not clear [88]. Posterior pituitary dysfunction—characterised by inappropriate secretion of antidiuretic hormone and cerebral salt wasting—is common after stroke. The cornerstones of treatment for cerebral salt wasting are fluid replacement and sodium administration; conversely, inappropriate secretion of antidiuretic hormone must be treated with fluid restriction. Hence, distinguishing the two and making the correct diagnosis is paramount to allow prompt initiation of proper treatment.

## Conclusions

Peripheral-organ dysfunctions are particularly common after ischaemic stroke, and their prevention and treatment usually require specific interventions. Knowledge of the nature and timing of systemic complications after acute ischaemic stroke, alongside the identification of high-risk patients, may be useful to allow prompt, early management of these patients and improve their outcomes. The most common complications after stroke include respiratory failure and cardiovascular dysfunction. Most peripheral-organ damage arises within the first week after stroke; however, there is little data to guide the management of these complications. *Additional*, carefully designed *studies* into the non-neurological complications of ischaemic stroke are warranted.

## Data Availability

Not applicable

## Abbreviations

MODS: Multiple organ dysfunction syndrome
ARDS: Acute respiratory distress syndrome
PBW: Predicted body weight
PEEP: Positive end-expiratory pressure
RM: Recruitment manoeuvres
FiO_2_: Fraction of inspired oxygen
ECG: Electrocardiogram
NIHSS: National Institute of Health Stroke Scale
AKI: Acute kidney injury
OR: Odds ratio
NAFLD: Non-alcoholic fatty acid disease
